# HEALTH AND WELL-BEING OUTCOMES OF MEALS ON WHEELS: A SYSTEMATIC REVIEW FOR A COMPLEX INTERVENTION

**DOI:** 10.1093/geroni/igae098.3847

**Published:** 2024-12-31

**Authors:** Bernadette Wright, Victoria Owens, Sanjaya Wilson

**Affiliations:** Meals on Wheels America, Arlington, Virginia, United States; Meals on Wheels America, Arlington, Virginia, United States; Meals on Wheels America, Arlington, Virginia, United States

## Abstract

Meals on Wheels programs deliver nutritious meals to older adults’ homes, providing the opportunity to address social and safety needs that affect health. To make informed decisions on programs and policies, decision-makers need to understand which Meals on Wheels services lead to specific impacts, identifying the affected populations, locations, and mechanisms involved. We investigated these questions through a systematic review of studies demonstrating Meals on Wheels’ effects on older adults’ health and health-related outcomes. Forty-eight publications met inclusion criteria, representing 47 studies and 105 outcome analyses. Outcomes frequently examined included diet quality (20), nursing home use/living independently (17), food insecurity (12), healthcare utilization (11), social connection (10), falls/home safety (6), nutrition (5), and other outcomes (24). Study designs included single group quasi-experiments (13), analyses of perceived outcomes (12), comparison group quasi-experiments (9), single-group pre-post studies (8), and randomized experiments (5). Majorities of studies were from the U.S. (38), in peer-reviewed publications (35), and published in the past 10 years (27). Risks of bias included unaccounted-for variables (11) and underpowered samples (9). Eighty-eight analyses (45 studies) found evidence of beneficial effect. Studies attributed success in part to meeting participant preferences and nutritional needs, serving those with greatest need, offering more meals/food, and ensuring opportunity for social connection. Seventeen analyses (9 studies) did not find positive effects, most frequently explained by methodological issues or participants not receiving enough services. These findings suggest that Meals on Wheels services that meet participants’ preferences and needs can reduce barriers to health and aging in place.

